# Exhaled nitric oxide decreases after positive food-allergen challenge

**DOI:** 10.1186/2045-7022-1-14

**Published:** 2011-11-28

**Authors:** Avigael H Benhamou, Alice Koehli, Isabelle Rochat, Demet Inci, Alexander Moeller, Philip Taramarcaz, Roger P Lauener, Philippe A Eigenmann

**Affiliations:** 1 Pediatric Allergy Unit, Geneva University Hospitals and University of Geneva, Geneva, Switzerland; 2 Pediatric Pulmonary Unit, Geneva University Hospitals and University of Geneva, Geneva, Switzerland; 3 Division of Allergology and Respiratory Medicine, University Children's Hospital Zurich, Zurich, Switzerland; 4 Centre des Allergies et de l'Asthme de la Terrassière, Geneva, Switzerland; 5 Christine Kühne-Center for Allergy Research and Education, Children's Allergy & Asthma Hospital, Hochgebirgsklinik Davos, Switzerland

**Keywords:** Allergen challenge, exhaled Nitric oxide, food allergy, food challenge

## Abstract

**Background:**

Exhaled nitric oxide (FeNO) is a well described marker of airway inflammation in asthma and is also known to increase after chronic exposure to inhaled allergens. It is not known whether monitoring FeNO could be useful during food challenges to detect early or subclinical reactions.

**Methods:**

Forty children aged 3 to 16 years undergoing an allergen-food challenge at two centres were prospectively recruited for this study. FeNO was assessed before and repeatedly after the food-challenge.

**Results:**

Data were obtained from a total of 53 challenges (16 positive, 37 negative) and were compared between the two groups. Half of the patients with a positive food challenge exhibited clinical upper respiratory symptoms. The FeNO significantly decreased in 7 of 16 patients with a positive challenge test within 60 to 90 minutes after the first symptoms of an allergic reaction.

**Conclusion:**

Our results show a significant decrease in FeNO after a positive food challenge suggesting involvement of the lower airways despite absence of clinical and functional changes of lower airways. Prospective blinded studies are needed to confirm these results.

## Introduction

The current prevalence of food allergy in childhood varies between 6-8% during the first 3 years of life [1]. Food challenges are the gold standard for the diagnosis, in particular when the patient's history and specific IgE test results do not correlate, as well as for follow-up assessment. Among other potential symptoms, lower respiratory symptoms can be elicited by a positive challenge; they constitute a major risk factor for severe life-threatening anaphylaxis. It has also been observed that up to 40% of children and adolescents with food allergy but without asthma have concomitant asymptomatic bronchial hyperreactivity (BHR) to methacholine, in general without lung function changes [2,3].

There is in agreement with the observation that fractional exhaled nitric oxide (FeNO) reflects bronchial allergic inflammation, and the measurement of FeNO has been proposed as a diagnostic tool for asthma [4], both in adults and children [5]. In addition, FeNO is related to the degree of bronchial inflammation in asthma and provides a non-invasive measure to monitor the anti-inflammatory treatment of asthmatic patients [6]. However it is unknown, whether FeNO changes occur early during food challenges in allergic children and if monitoring FeNO could serve to detect positive responses during such an allergen challenge.

It has been shown earlier that FeNO increases after specific bronchial allergen provocation in adults [7], whereas no changes in FeNO have been reported after a nasal allergen challenge in children [8]. More recently, no change in FeNO was found during a milk food challenge in infants [9].

We hypothesised, that a food-induced, IgE-mediated allergic reaction might provoke a modification in FeNO during or early after food challenges and hence, FeNO may serve as an early objective marker of a positive reaction involving the respiratory tract.

## Materials and methods

Patients were recruited from two separate paediatric allergy centres in a prospective study with the primary aim of measuring FeNO changes in children undergoing food challenges. All data were treated confidentially and available only to the investigating physicians. The study was reviewed and approved by the Ethics Committee of the Department of Paediatrics of the participating institutions and was subjected to patient and/or parental written consent.

### Patients

Children were recruited at the Paediatric Allergy Clinics at the Geneva University Hospital and at the University Children's Hospital Zurich, Switzerland between May 2007 and March 2009. Inclusion criteria were children between 3 to 16 years of age, with or without a history of asthma, undergoing a diagnostic or follow-up oral food-allergen challenge. Exclusion criteria were any asthma treatment current or within the previous four weeks (oral or inhaled steroids, inhaled bronchodilators and/or leukotriene receptor antagonists), or a history of an acute respiratory tract infection in the previous four weeks. Sensitisation to common aeroallergens (grass and tree pollen, animal dander, dust mites and moulds) was determined by skin prick tests with commercially available extracts (Allergopharma, Reinbek, Germany), and/or by specific IgE measurement by UniCAP™ System (Thermo Fisher Scientific, Uppsala, Sweden).

### Food challenges

Oral food challenges were performed according to previously published guidelines [10], for diagnosis in patients with a suspicion of IgE-mediated food allergy, or for food allergy follow-up in children previously diagnosed. Two different challenge protocols were used according to the clinical history. Patients with immediate-type reactions were tested by an open food challenge, and those with atopic dermatitis or potentially presenting with subjective, but immediate-type reactions were tested by double-blind, placebo-controlled food challenges (DBPCFC), with the two challenges (placebo and tested food) done the same day, with a free interval of minimum 2 hours and only if the morning challenge was negative. Oral anti-histamines were stopped at least 5 days before the challenge test. The challenged food was administered in five increasing doses every 15 to 30 minutes. Test foods were milk, hen's egg, sesame, hazelnuts and tree nuts, peanuts, soy, fish, wheat, and mushroom. The challenge test was stopped, when a) there was a convincing clinical reaction (positive test), such as an urticarial rash, laryngeal symptoms, vomiting, a marked decreased activity, an acute rhinitis or cough, wheezing or chest tightness, as described in the guidelines [10] or b) when the final dose of the test was achieved without a clinically subsumable reaction (negative test). In children with a positive test, the reaction was graded from 1 [very mild] to 5 [anaphylactic shock] [11], and a treatment was administered according to the severity of the symptoms [12]. All children were observed for at least 2 hours after the end of the challenge test.

### Measurement of FeNO

Exhaled nitric oxide (FeNO) was measured in all patients before the food challenge, every 30 minutes during the 2 hours observation period, and after the completion of the food challenge. According to American Thoracic Society/European Respiratory Society recommendations [13], FeNO was collected in a single breath offline collector system with controlled expiratory flow and NO filter and analyzed in a chemiluminescence analyser (CLD77TM; Ecomedics, Duernten, Switzerland). A change of FeNO of more than 6 ppb from baseline was considered as significant ^, ^as previous studies have shown that FeNO measurement where consistent within 0.5 ppb of the results using chemiluminescence technique [14] and that FeNO results for a same volunteer are reproducible within 2 ppb, with no diurnal variation or learning effect [15].

### Statistical Analysis

Comparison of FeNO levels in children with positive or negative challenge tests were analysed by a non-parametric test (Wilcoxon rank-sum test). The in-patient difference from the sequentially measured FeNO values (at 30, 60, 90 or 120 min) and the baseline FeNO [FeNOt-FeNOb] were analysed by Wilcoxon signed-rank test. A Fisher's exact t-test was used for in-between patient group analysis. Pulmonary function test results (pre-test compared to post-test in the same patient) were analyzed by the t test for paired samples. Data are presented as median and ranges, or as mean with standard deviations when normally distributed. Differences were considered to be statistically significant when the p-value was <0.05.

## Results

### Food challenges

Fifty-three food challenges were performed in 40 children (18 girls, 22 boys). Eleven children underwent 2 or 3 challenges, because of a double-blind, placebo-controlled procedure or because 2 different foods were tested. No challenge was performed the same day after a positive food challenge. Sixteen of the challenges were positive, in fifteen patients (16/53, 30%) (Table 1), and 1 patient had two positive challenges, with raw egg and peanuts, done at a 5 months interval. All patients presented clinical reactions interpreted as mild to moderate [11]. Among these patients, eight had respiratory symptoms such as nasal congestion, sneezing, rhinorrhea, sensation of throat tightness. None of the patients exhibited a severe respiratory reaction with overt symptoms of the lower respiratory tract (no coughing, wheezing, dyspnoea and/or cyanosis). Non-respiratory symptoms in the remaining positive challenges included cutaneous or gastro-intestinal reactions, and/or a marked decreased activity.

**Table 1:** Characteristics of patients with a positive food challenge

Age (y.m)	Gender	Food	Food-specific IgE titres (kU/L)	Reaction Score (1 [very mild] to 5 [anaphylactic shock]*)	Respiratory reaction (as defined in the text)	Decreased FeNO at 60 minutes(>6 ppb)	Decreased FeNO at 90 minutes (>6 ppb)
9.9	F	Peanut	0.95	2	Y	Y	Y
9.8	F	Peanut	8.28	3	Y	Y	N
8.6	M	Peanut	1.07	1	N	N	N
10.1	F	Peanut	18.6	2	N	N	Y
6.8	M	Peanut	31.6	3	Y	Y	Y
6.9	M	Peanut	2.93	3	Y	Y	Y
6.5	M	Peanut	6.71	2	N	N	N
5.1	M	Milk	4.75	2	N	N	N
5.1	F	Hazelnut	7.04	2	N	N	Y
3.5	M	Hazelnut	<0.35	1	N	N	N
7.1	M	Tree nut	2.50	3	Y	N.A.	N.A.
5.1	F	Tree nut	1.91	3	Y	Y	Y
9.2	F	Raw egg	1.58	2	Y	N	N
9.9	F	Raw egg	2.07	2	N	Y	Y
5.7	F	Boiled egg	2.92	2	N	Y	N.A.
7.5	F	Boiled egg	8.6	2	Y	N	N

M: male, F: female, Y: yes, N: no

* according to (11).

### Allergy tests

Sensitisation to common aeroallergens was assessed by skin pricks tests and/or specific IgE measurements. The tests were positive to one or more aeroallergens in 14/15 (93%) patients with positive food challenges, and in 21/28 (75%) of those with negative challenges (p = n.s).

### FeNO

**Figure 1:**
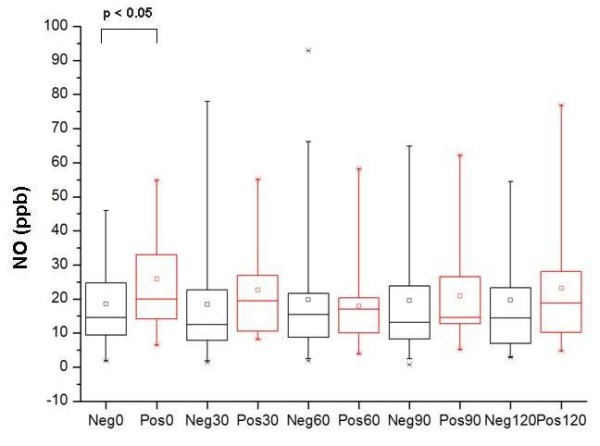
**FeNO measurements in ppb before (0 min), and 30, 60, 90 and 120 minutes after the last dose of a food-antigen challenge**. The results are grouped at the different time points by food-antigen challenge results, negative (Neg0, Neg30, Neg60, Neg90 or Neg120) or positive (Pos0, Pos30, Pos60, Pos90 or Pos120), and plotted as median value with lower and upper quartiles.

Median pre-challenge FeNO values were higher in patients with positive food challenges (median 20.7 ppb [range 6.4-54.8]) compared to those with negative challenges (median 14.5 ppb [range 1.8-109.3]) (p = 0.04) (Figure 1). At different time points after the challenge (30, 60, 90 and 120 minutes, respectively) there were no significant differences in FeNO between children presenting with or without a positive challenge test.

**Figure 2:**
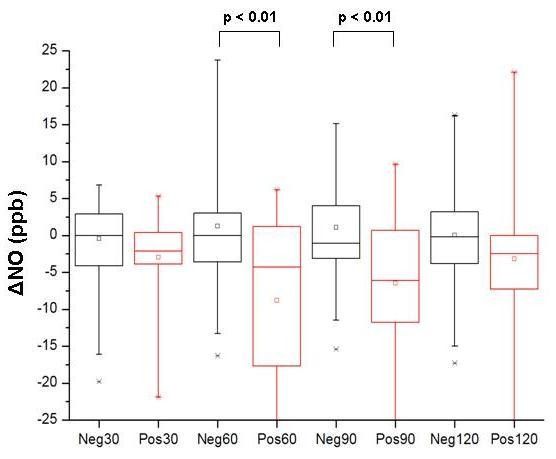
**FeNO change from baseline (ΔNO) at 30, 60, 90 and 120 minutes after the last dose of a food-antigen challenge**. The results are grouped by food-antigen challenge results, negative (Neg30, Neg60, Neg90 or Neg120) or positive (Pos30, Pos60, Pos90 or Pos120), and plotted as median value with lower and upper quartiles.

When pre-challenge FeNO measurements were compared to sequential measurements after the food challenges a significant decrease in FeNO was measured in positive patients at 60 and 90 minutes (Figure 2). When a change of FeNo superior of 6 ppb was considered, a significant change was observed in 3 out of 16 (19%) of children with a positive challenge at 30 minutes after the completion of the test (p = 1), in 7/15 (47%) children (p = 0.003) and 7/14 (50%) children (p = 0.005) after 60 and 90 minutes, respectively. After 120 minutes a decrease of > 6 ppb from baseline was still observed in 5 of 15 (33%) patients, however without statistical significance (p = 0.08) (Table 2). Neither pre-challenge FeNO levels, nor FeNO changes did correlate to the clinical severity of the allergic reaction during the challenge test. The type of clinical reaction to the challenge test, respiratory or non-respiratory was not associated with changes in FeNO.

**Table 2:** Number of food challenges with or without a significant decrease in FeNO (> 6 ppb)

Time after last antigen dose	30 min		60 min		90 min		120 min	
**Outcome of food challenge**	**Pos**.	**Neg**.	**Pos**.	**Neg**.	**Pos**.	**Neg**.	**Pos**.	**Neg**.
ΔFeNO >6 ppb	3	5	7	4	7	4	5	2
No change in FeNO	13	30	8	33	7	33	10	31

P=	1 (n.s.)		0.003		0.005		0.08 (n.s.)	

Pos.: positive food challenge; Neg.: negative food challengen.s.: non significant

**Figure 3:**
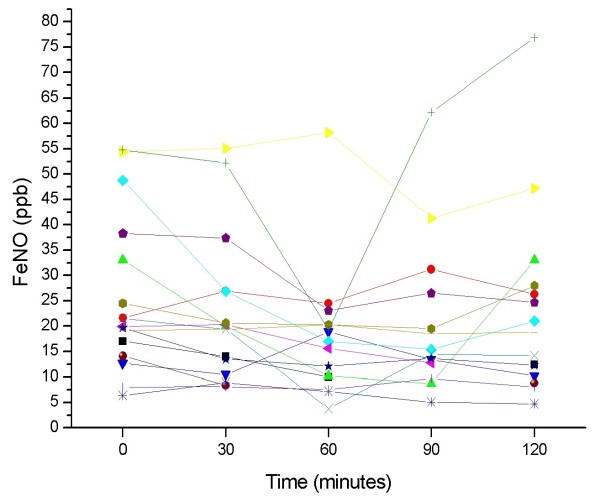
**Individual changes in FeNO values measured throughout the test in patients with a positive food-antigen challenge**.

**Figure 4:**
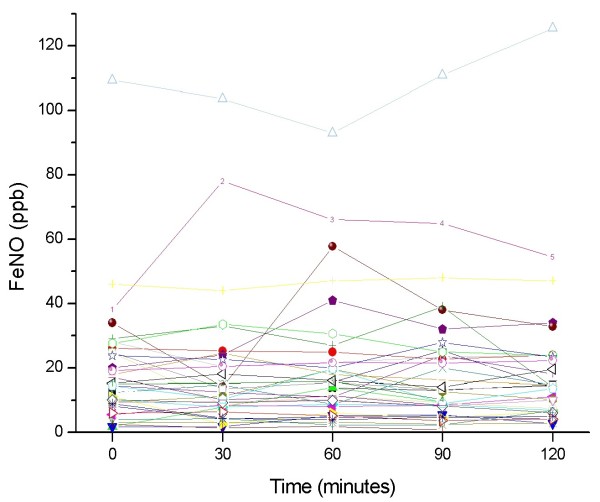
**Individual changes in FeNO values measured throughout the test in patients with a negative food-antigen challenge**.

In patients with negative food challenges, there was no significant change in FeNO throughout the observation period, with variations of more than 6 ppb (increase or decrease) observed only in 5 of 36 patients (14%) at 30 minutes, 4 of 37 patients (11%) at 60 and 90 minutes, and 2 of 33 patients (6%) at 120 minutes post challenge. Individual changes in FeNO throughout the test are presented in Figure 3 (positive challenges), and Figure 4 (negative challenges).

## Discussion

It is well recognized that respiratory reactions may potentially lead to life-threatening reactions of food allergy. In this study, we investigated whether measurement of FeNO was useful for early detection of reactions during food challenges. A significant number of positive food-allergen challenges were linked to a decreased FeNO, when compared to baseline, at 60 and 90 minutes after the first symptoms of allergy, in patients with and without objective lower respiratory tract symptoms. These changes were seen in patients regardless of objective lower respiratory tract symptoms. No specific patient characteristics (e.g. age, food challenged or type of symptoms) were linked to FeNO changes.

Convincing evidences indicate that allergic reactions to foods may, in addition to clinical reactions, also elicit local inflammation with a direct consequence on functional tests. An early decrease in lung function parameters has been previously described after respiratory allergen challenge [16]. In addition, increased bronchial hyperreactivity demonstrated by a methacholine challenge before and after a food challenge may occur despite a normal FEV_1 _immediately after the food challenge [17]. These two studies clearly showed that allergen-induced allergic reactions can induce airway changes, without lower respiratory tract symptoms in some patients, and that airway inflammation during allergen challenges is probably largely unrecognized. FeNO in relation to food allergy has so far only been studied in infants with milk allergy [9]. No variation in FeNO could be observed, although most patients suffered from a late-type allergic reaction, probably not with an IgE-mediated mechanism.

Late increase of FeNO after nasal or bronchial allergen challenge or after natural allergen exposure [18], suggests that FeNO may also increase similarly after a positive food-allergen challenge. All our patients with a positive challenge experienced an early, IgE-type allergic reaction, and a significant part of these patients showed, within 60 to 90 minutes after the clinical reaction, a decrease in FeNO, which is probably in relation with the early phase of inflammation. There were no significant changes of FeNO in children with negative food challenges. Khatri et al. have reported temporal association of nitric oxide levels after whole lung allergen challenge and observed, similarly to us, that FeNO levels decreased significantly from baseline, between 2 to 5 hours after the challenge, and returned to normal levels at 24 hours. They hypothesized that the FeNO decrease first observed might be possibly due to consumption by reactive oxidant species into peroxynitrite [16]. Similarly, Pedroletti et al. observed a decrease in FeNO 6 hours after an allergen nasal challenge in asthmatic children [8]. However, this change was similar in the non-allergic control group and no increase was observed in either group after 24 h. The authors suggested that the single dose nasal challenge might not have been potent enough to induce a prolonged inflammation. Previous studies in which FeNO was increasing mainly explored FeNO changes in late phase reactions in airway allergen challenges [4,19,20], while our patients were tested during the early phase of inflammation. A secondary increase in FeNO would have been possible in our patient population, but could not be measured here due to the food challenge schedule with a discharge 2 hours after completion of the procedure.

It should be noted that our study suffers from some limitations, inherent to the food challenges. Due to the various foods tested and to individual responses in positive food challenges, it is not possible to have a "standard" positive responder. However, the values were decreasing in all patients, in whom a modification in FeNO was observed, constantly indicating a change towards the same direction. In addition, no other markers of inflammation have been measured, thus not allowing further elaborating on potential mechanisms for FeNO changes.

In summary, we could show an early decrease in FeNO between 60 and 90 minutes after a positive food allergen challenge, regardless of objective respiratory symptoms. Our study clearly demonstrates that IgE-specific allergic reactions to foods are involving the lung, with FeNO changes observed also in absence of overt clinical respiratory symptoms. Our study does not provide an explanation for the decreased FeNO measures after positive food allergen challenges; further studies should explore our current findings.

## Abbreviations

FeNO: Fractional Exhaled Nitric Oxide; BHR: Bronchial Hyperreactivity.

## Competing interests

The authors declare that they have no competing interests.

## Authors' contributions

AHB participated in the study coordination, contributed to the recruitment of patients, data acquisition and analysis and manuscript drafting. AK participated in the study design and data acquisition and analysis. IR participated in the study design and coordination and helped to draft the manuscript. DI participated in data acquisition and revising the manuscript. AM participated in the study design and coordination and helped to draft the manuscript. PT participated in the study design and coordination. RL participated in the study design and coordination and revising the manuscript. PAE participated in the study design and coordination, contributed to the recruitment of patients, data analysis and manuscript revising. All authors read and approved the final manuscript.
